# Peer Review of “Medical Expectations of Physicians on AI Solutions in Daily Practice: Cross-Sectional Survey Study”

**DOI:** 10.2196/56496

**Published:** 2024-03-25

**Authors:** Francesco Baglivo

**Affiliations:** 1 Department of Translational Research and New Technologies in Medicine and Surgery, University of Pisa, Pisa, Italy

**Keywords:** artificial intelligence, adoption, acceptance, opinion, perceptions, survey, expectations, physician, medical survey, qualitative study, AI

*This is the peer-review report for “Medical Expectations of Physicians on AI Solutions in Daily Practice: Cross-Sectional Survey Study.”*

## Round 1 Review

### General Comments

The manuscript [1] delves into the perspectives of Brazilian physicians on the integration of artificial intelligence (AI) in medical practices through an online cross-sectional survey.

### Specific Comments

#### Major Comments

1. The study purports to evaluate the acceptance of AI by physicians, but the specific types of AI technologies explored remain ambiguous. Are they examining generative AI, natural language processing tools, classical machine learning, or other uses of AI?
2. The phrase “Although scarcely used in real practice” comes across as too assertive. Consider a softer phrasing.
3. The methods section should provide a more comprehensive breakdown of the questionnaire’s design process. Which question types were chosen (Likert scale, yes or no, or numerical), and for what reasons?
4. When presenting results, always give raw data (numerator/denominator) along with percentages, especially after statements such as “Most of them described their AI knowledge as intermediate.”
5. There is a noticeable omission of a power analysis. How can we ascertain that the sample size sufficiently represents the broader population? The description of the target population needs elaboration.

#### Minor Comments

1. The statement “Artificial intelligence (AI) applied to Medicine has been a trending subject in recent years” is preferable over mentioning it as the “hottest topic.”
2. In Table 1, the age bracket should read “50-65” as the “50” seems to be missing. Several *P* values appear without context, for instance: “10. General AI Knowledge (n=164); *P*=.2565,” “11. Regularity of AI tool usage in daily life (n=164); *P*=.9792,” and “12. Familiar with medical AI solutions? (n=164); *P*=.2774.”
3. Tables 2 and 3 also contain *P* values that require explanations or clarifications.

## Abbreviations

AI: artificial intelligence
